# Advances in Pediatric and Adult Cochlear Implant and Middle Ear Prostheses

**DOI:** 10.3390/jcm10143152

**Published:** 2021-07-16

**Authors:** Andrea Ciorba, Piotr H. Skarżyński, Hulya Gocmenler, Stavros Hatzopoulos

**Affiliations:** 1 Audiology and ENT Unit, Department of Neurosciences and Rehabilitation, University Hospital of Ferrara, University of Ferrara, 44224 Ferrara, Italy; sdh1@unife.it; 2 Institute of Sensory Organs, Kajetany, 05-830 Warsaw, Poland; piotrhskarzynski@gmail.com; 3 Institute of Physiology and Pathology of Hearing, 05-830 Warsaw, Poland; 4 Department of Heart Failure and Cardiac Rehabilitation, Medical University of Warsaw, 02-091 Warsaw, Poland; 5 Department of Audiology, Faculty of Health Sciences, Istanbul Medeniyet University, Istanbul 34720, Turkey; gocmenler.hulya@gmail.com

## 1. About this Volume

The aim of this special issue, entitled ‘Advances in Pediatric and Adult Cochlear Implant and Middle Ear Prostheses’, is to report the *undergoing novel research* in the area of ear prostheses, either for the middle or inner ear. The current trends are promising exciting clinical applications within 5–10 years. Local inner ear therapy via a hearing prosthesis has already been realized and alternative stimulus optimization protocols are being developed. Special attention is being paid in the area of pediatric intervention since the quality of life of young patients is always a highly considered factor.

## 2. Introduction

Hearing loss is reported to be among the most prevalent disabilities in Western countries, as it has been estimated to involve up to 30% of the adult population [1,2,3]. According to the most recent estimates, about 70 million people have received a diagnosis of severe hearing loss and it was reported that by 2050, approximately 900 million people will be affected by age-induced hearing losses [1,2,3].

The restoration of hearing and the rehabilitation strategies for hearing loss are based on the data from the correct identification of the causes that affect the auditory system and determine the treatment possibilities. Modern technology offers numerous solutions, including the rehabilitation and treatment of cases that present moderate, severe or profound sensorineural hearing impairment. Currently, hearing impairment is overcome by technological solutions that are related to hearing aids (HAs), implantable hearing aids, cochlear implants (CIs) and, more rarely, auditory brainstem implants.

Cochlear implants are considered among the most effective neural prostheses ever developed. Presently, CI technology is further evolving at a great pace in terms of hardware and software solutions. From the single-channel processors used in the early 1960s, the CI devices are nowadays equipped with multiple channels, allowing for the stimulation of multiple cochlear locations. The CI stimulus processing strategies have also evolved, targeting a higher performance index regarding auditory pathway activation [4,5,6,7].

The paragraphs below summarize the most distinct current trends and underline what is expected in the near future in the field of hearing prostheses and hearing restoration.

A. *Size and connectivity optimization*: Future steps could be directed toward the optimization of the size/volume and the increased connectivity of the hearing restoration device. Intelligent connectivity is considered to be a trend in vogue since there are numerous advantages to the users when CIs and HAs can interact intelligently with other everyday life devices (smartphones, smart cars and smart TVs via dedicated apps). Currently, CI and HA users can moderately interact with their smartphones using Bluetooth-dedicated channels. Interesting ideas within this field include the possibilities of self-fitting a device using a smartphone. The implications from such a technological step are vast and, in combination with advances in teleaudiology, can result in an affordable rural assistance model [8,9].

B. *Local ear therapy*: This aspect is mostly related to CIs but could eventually be expanded to middle-ear-implantable devices. It is likely that in the near future, CIs may undergo several structural changes from using normal electrodes to electrodes containing films or reservoirs that release agents that can perform local cochlear therapy (intra-cochlear administration of drugs). In this context, it could be possible to even further enhance auditory rehabilitation, for example, by releasing local growth factors/neurotrophins targeting specific spiral ganglion neurons. This approach could boost a CI’s neural performance by stimulating the neuronal response to electric stimulation; on the other hand, it could allow for the protection of the inner ear structures from cochlear mechanical trauma that is caused by the insertion of the CI electrodes [4,5,6,7,10].

C. *Globalization of the prostheses solution*: In order to offer the above-mentioned hearing restoration and rehabilitation solutions on a larger global scale, a reduction in the total costs should be attained within the near future [11]. Indirect technological indices suggest that hearing prostheses’ costs might drop, as the costs of advanced technological components tend to decrease. Unfortunately, software solution costs tend to increase as systems become more complex since they require a higher level of control. The balance between the hardware and software characteristics will probably define the final hearing prostheses’ costs in the near future.

## 3. General Rehab Solutions for Hearing Loss

Concerning other restoration and rehabilitation solutions for hearing losses that are related to physical damage to the external or the middle ear, there are several interesting fields of research that could possibly yield new clinical solutions in the future. For instance, bioactive materials were proposed by several authors for improving the adaptation or resolution of cartilage or bony tissue defects [12,13]. In this context, it is possible that tailored bio-scaffolds, in combination with cell cultures and growth factors, could be used, for instance, for a personalized reconstruction of an auricular pinna or the restoration of other specific local defects. This technology could also involve the middle ear, as new ossicular chain prostheses could be developed with new (and more bio-compatible) materials.

Finally, the application of nanomedicine and nanotechnologies could offer further solutions for the treatment of hearing disorders. In fact, nanoparticles (NPs) were proposed as carriers of various drugs for local inner ear therapy. Ideally, NPs should have a series of specific features, such as the capacity to permeate the round or oval window membrane, the ability to identify specific targets or the controlled release of their therapeutic loads in a safe way [14,15,16,17]. In these contexts, several authors have described attempts for the local delivery (via dedicated pumps or via a hearing prosthesis) of drugs or growth factors in mice or guinea pigs with variable results so far [14,15,16,17]. Although much progress still has to be done, this field of research seems very promising.
